# Association between immunity and viral shedding duration in non-severe SARS-CoV-2 Omicron variant-infected patients

**DOI:** 10.3389/fpubh.2022.1032957

**Published:** 2022-12-22

**Authors:** Shaojun He, Yanhong Fang, Jiong Yang, Wei Wang

**Affiliations:** ^1^Department of Respiratory and Critical Care Medicine, Zhongnan Hospital of Wuhan University, Wuhan, China; ^2^Department of Respiratory Medicine, Shanghai New International Expo Center Fangcang Shelter Hospital, Shanghai, China

**Keywords:** coronavirus disease 2019, Omicron variant, prolonged elimination, lymphocytes, eosinophils, albumin

## Abstract

**Background:**

Coronavirus disease 2019 (COVID-19) is a respiratory-related disease caused by severe acute respiratory syndrome coronavirus 2 (SARS-CoV-2). More than 200 countries worldwide are affected by this disease. The Omicron variant of SARS-CoV-2 is the major epidemic variant worldwide and is characterized by higher infectivity. However, the immunity and risk factors for prolonged viral elimination in patients with non-severe SARS-CoV-2 Omicron variant infections are unclear. Therefore, this study aimed to examine the relationship between immunity and duration of viral elimination in non-severe SARS-CoV-2 Omicron variant-infected patients in Shanghai.

**Methods:**

In total, 108 non-severe SARS-CoV-2 Omicron variant-infected patients from Shanghai New International Expo Center Fangcang Shelter Hospital were recruited in this study. They were further allocated to the early elimination (EE) and prolonged elimination (PE) groups according to SARS-CoV-2 nucleic acid positivity duration.

**Results:**

Compared to patients with EE, those with PE had increased serum concentrations of interleukin (IL)-5, IL-6, and IL-8; higher neutrophil count and neutrophil-to-lymphocyte ratio (NLR); lower lymphocyte, eosinophil, and red blood cell counts; and lower concentrations of hemoglobin and albumin (ALB). In lymphocyte subpopulation analysis, lower numbers of CD3^+^ T cells, CD4^+^ T cells, CD8^+^ T cells, and NK cells and a higher CD4/CD8 ratio were observed in patients with PE. In addition, correlation analysis results revealed that cycle threshold values of SARS-CoV-2 Omicron variant ORF1ab and N were negatively correlated with IL-6 and IL-8 levels and positively correlated with eosinophil count in patients with COVID-19. Finally, multivariate regression analysis showed that ALB, CD4/CD8 ratio, NLR, and eosinophil count were predictors of the SARS-CoV-2 Omicron variant elimination.

**Conclusion:**

In this study, we identified that the ALB, CD4/CD8 ratio, NLR, and eosinophil count were risk factors for prolonged viral elimination in non-severe SARS-CoV-2 Omicron variant-infected patients. These factors might be efficient indicators in the diagnosis, evaluation, and prognosis monitoring of the disease.

## Introduction

Coronavirus disease 2019 (COVID-19), a new type of pneumonia caused by severe acute respiratory syndrome coronavirus 2 (SARS-CoV-2), was first detected in Wuhan, China in December 2019 (1). At the time of writing, the number of COVID-19 confirmed cases totaled 551 million, with 6.35 million deaths reported to the WHO (2). The COVID-19 pandemic has strained healthcare systems worldwide (3). The SARS-CoV-2 Omicron variant, first reported on November 25, 2021, has become the dominant epidemic strain worldwide (4). The Omicron variant is significantly more transmissible than other variants of SARS-CoV-2 (5, 6), which poses a more significant challenge for epidemic control.

In late February 2022, an outbreak of the Omicron strain of COVID-19 was reported in Shanghai, China (7). During the Omicron variant epidemic in Shanghai, most infections were non-severe, and the viral shedding duration was within 10 days. However, a small number of non-severe infected patients took more than 10 days or even more than a month to clear the virus. The SARS-CoV-2 cycle threshold (Ct) value is negatively correlated with viral load and reflects the infectivity and duration of viral elimination to some extent (8, 9). Moreover, the immune response in patients with COVID-19 is related to the severity and outcome (10). However, limited studies have focused on the immune status and risk factors for prolonged viral elimination in patients with COVID-19. Thus, this study aimed to examine the relationship between immunity and duration of viral elimination in non-severe SARS-CoV-2 Omicron variant-infected patients in Shanghai.

## Materials and methods

### Patient population

**Figure 1:**
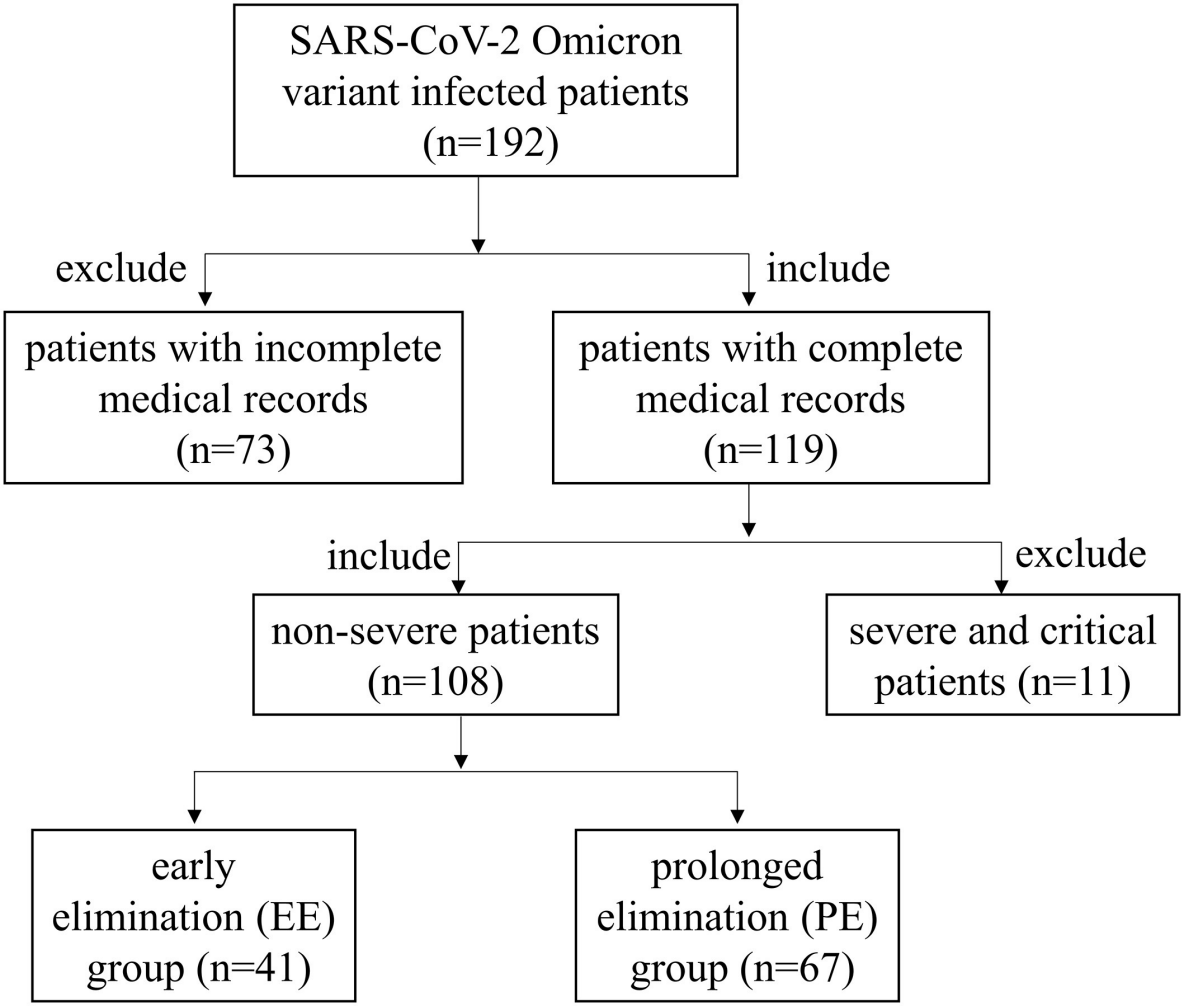
Screening process for 108 patients in this research.

The Ethics Committee of Zhongnan Hospital of Wuhan University approved this retrospective study protocol (No. 2022109K) and waived the requirement for written informed consent from patients. This study included 108 patients diagnosed with COVID-19 caused by the SARS-CoV-2 Omicron variant based on nucleic acid detection of the N and ORF1ab genes. The screening process for 108 patients was shown in Figure 1. All patients were admitted to Shanghai New International Expo Center Fangcang Shelter Hospital between April 20, 2022 and May 31, 2022.

Disease severity was defined according to the guidelines of the Chinese National Health Commission for SARS-CoV-2 (Trial Version 9) as follows: (a) asymptomatic, with SARS-CoV-2 Omicron variant nucleic acid positivity, but no clinical symptoms and no pneumonia presentation on radiologic images; (b) mild, with slight clinical symptoms but no pneumonia presentation on radiologic images; (c) moderate, with clinical symptoms including fever and respiratory tract involvement and pneumonia presentation on radiologic images; (d) severe, with any of the following conditions: shortness of breath, respiratory frequency ≥30 times/min; finger oxygen saturation ≤93% at rest; arterial oxygen tension/inspiratory oxygen fraction (PaO_2_/FiO_2_) ≤300 mmHg; progressive exacerbation of clinical symptoms, pulmonary infiltrates on radiologic images >50% of lung volume within 24–48 h; and (e) critical, with any of the following conditions: respiratory failure requiring mechanical ventilation, shock, and other organ failures requiring intensive care and treatment.

The criteria for discharge from the hospital were as follows: (a) afebrile for >3 days; (b) improved respiratory symptoms; (c) radiological examinations showing obvious absorption of lung lesions; and (d) negative nucleic acid tests (both ORF1ab and N gene Ct value ≥35) twice consecutively (sampling interval ≥24 h) (11).

Asymptomatic, mild, or moderate cases were considered non-severe. Patients with non-severe COVID-19 who tested SARS-CoV-2 positive within 10 days after diagnosis were allocated to the early elimination (EE) group and those who tested SARS-CoV-2 positive for more than 10 days were allocated to the prolonged elimination (PE) group (12, 13).

### Data collection

Data on the following patient characteristics were collected: age, sex, clinical manifestations, underlying diseases, and vaccine inoculation. Blood routine tests included tests for estimating white blood cell (WBC), neutrophil, lymphocyte, monocyte, eosinophil, basophil, platelet (PLT), and red blood cell (RBC) counts; neutrophil-to-lymphocyte ratio (NLR); and hemoglobin (Hb) concentration. Liver function tests included those for estimating albumin (ALB), alanine aminotransferase (ALT), aspartate aminotransferase (AST), alkaline phosphatase (ALP), glutamyl transpeptidase (GGT), and total bilirubin concentrations. Previous studies have reported dysregulated adaptive immune responses in patients with COVID-19 (1). However, changes in lymphocyte subsets in non-severe Omicron variant-infected patients have not been examined. Therefore, we further analyzed each lymphocyte subpopulation's absolute numbers and relative frequencies in the EE and PE groups. Lymphocyte subpopulation tests included those for estimating the frequency and absolute numbers of B cells, CD3^+^ T cells, CD4^+^ T cells, CD8^+^ T cells, NK cells, and CD4/CD8 ratio, were examined by FACS Aria III cytometer (BD bioscience, USA). Serum cytokines, including interleukin (IL)-2, IL-4, IL-5, IL-6, IL-8, IL-10, IL-12p70, IL-17A, tumor necrosis factor (TNF)-α, interferon (IFN)-α, and IFN-γ, were measured using a Multi-Analyte Flow Assay Kit (Biolegend, USA). SARS-CoV-2 viral nucleic acid was detected using real-time reverse transcription-polymerase chain reaction assays and presented as Ct values of the ORF1ab and N genes. To identify biomarkers that might be useful in diagnosing COVID-19, we compared the Ct values of the COVID-19 ORF1ab/N gene with laboratory findings that were significantly different between the EE and PE groups.

### Statistical analysis

Continuous variables are presented as median [interquartile range (IQR)]. Normally distributed variables were compared using Student's *t*-test, and non-normally distributed continuous variables were compared using the Mann–Whitney test. Categorical variables, presented as numbers (percentages), were compared using the chi-square test or Fisher's exact test. The relationship between SARS-CoV-2 ORF1ab/N gene Ct values and laboratory findings was examined using Spearman's rank correlation test. Univariate and multivariate logistic regression analyses were performed to determine the significant predictors of prolonged viral elimination. Data analysis was performed using SPSS software (version 22.0; IBM SPSS Statistics, IBM Corporation), and statistical significance was set at *p* < 0.05.

## Results

### Baseline characteristics

After initial screening, 108 patients with non-severe COVID-19 admitted to Shanghai New International Expo Center Fangcang Shelter Hospital were enrolled in this study. The median age of all patients was 52.5 years, and 28.70% of patients were aged ≥65 years. Further, 51.9% of patients were men. The most common symptoms were cough (26.85%), fever (22.22%), expectoration (10.19%), and fatigue (10.19%). The common complications included hypertension (15.74%), diabetes (7.41%), cardiovascular diseases (2.78%), cerebrovascular disease (2.78%), and chronic obstructive pulmonary disease (2.78%). Vaccine inoculation showed that 76.07% of patients received at least one COVID-19 vaccine dose and 43.52% received a third booster shot. Patients in the PE group were older than those in the EE group by a median of 7 years (49 vs. 56 years, *p* = 0.0042). Diarrhea was more common in patients with EE than in those with PE (9.76 vs. 1.49%, *p* = 0.048). The detailed patient characteristics are presented in Table 1.

**Table 1:** Baseline characteristics of COVID-19 patients.

**Characteristics**	**Total (*N =* 108)**	**EE group (*N =* 41)**	**PE group (*N =* 67)**	***p*** -value^*^
**Sex**				
Male	56 (51.85%)	27 (65.85%)	29 (43.28%)	**0.023**
Female	52 (48.15%)	14 (34.15%)	38 (56.72%)	
**Age (years) median (IQR)**	52.5 (42.0–65.8)	49 (36–58)	56 (47–71)	**0.0042**
**Age group (years)**				
0–18	2 (1.85%)	1 (2.44%)	1 (1.49%)	0.617
19–49	41 (37.96%)	19 (46.34%)	22 (32.84%)	0.16
50–64	34 (31.48%)	14 (34.15%)	20 (29.85%)	0.641
≥65	31 (28.70%)	7 (17.07%)	24 (35.82%)	**0.037**
**Symptoms**				
Cough	29 (26.85%)	10 (24.39%)	19 (28.36%)	0.652
Fever	24 (22.22%)	9 (21.95%)	15 (22.39%)	0.958
Expectoration	11 (10.19%)	3 (7.32%)	8 (11.94%)	0.443
Fatigue	11 (10.19%)	3 (7.32%)	8 (11.94%)	0.443
Pharyngalgia	6 (5.56%)	4 (9.76%)	2 (2.99%)	0.138
Diarrhea	5 (4.63%)	4 (9.76%)	1 (1.49%)	**0.048**
Dizziness/headache	4 (3.70%)	1 (2.44%)	3 (4.48%)	0.588
Nasal obstruction	2 (1.85%)	0 (0.00%)	2 (2.99%)	0.264
Chest tightness	1 (0.93%)	0 (0.00%)	1 (1.49%)	0.434
Others	1 (0.93%)	0 (0.00%)	1 (1.49%)	0.434
Asymptomatic	57 (52.78%)	25 (60.98%)	32 (47.76%)	0.184
**Comorbidities**				
Any	30 (27.78%)	5 (12.20%)	25 (37.31%)	**0.005**
Hypertension	17 (15.74%)	4 (9.76%)	13 (19.40%)	0.182
Diabetes	8 (7.41%)	1 (2.44%)	7 (10.45%)	0.125
Cardiovascular diseases	3 (2.78%)	0 (0.00%)	3 (4.48%)	0.171
Cerebrovascular disease	3 (2.78%)	2 (4.88%)	1 (1.49%)	0.301
COPD	3 (2.78%)	0 (0.00%)	3 (4.48%)	0.171
Others	6 (5.56%)	1 (2.44%)	5 (7.46%)	0.271
**Coronavirus vaccination**				
0	28 (25.93%)	9 (21.95%)	19 (28.36%)	0.463
1	2 (1.85%)	1 (2.44%)	1 (1.49%)	0.725
2	30 (27.78%)	10 (24.39%)	20 (29.85%)	0.539
3	47 (43.52%)	21 (51.22%)	26 (38.81%)	0.207
**Ct value**				
ORF1a/b	35.75 (30.17–38.12)	37.88 (33.62–39.00)	34.11 (28.97–37.94)	**0.0413**
N	35.03 (28.82–38.48)	37.34 (34.19–38.99)	33.00 (28.34–37.32)	**0.0182**

Data are presented as median (IQR) or n (%).

* *p*-value indicates differences between EE and PE patients. *p* < 0.05 is considered statistically significant (in bold). COVID-19, coronavirus disease 19; Ct, cycle threshold; EE, early elimination; PE, prolonged elimination.

### Alteration of cytokine expression in patients with non-severe COVID-19

The cytokine concentrations in the serum are presented in Table 2. The concentrations of most cytokines, including IL-1β, IL-2, IL-4, IL-10, IL-12p70, IL-17A, TNF-α, IFN-α, and IFN-γ, were unaltered between patients with EE and PE. However, IL-5 (*p* = 0.0023), IL-6 (*p* = 0.0394), and IL-8 (*p* = 0.0042) concentrations were significantly increased in the PE group than in the EE group.

**Table 2:** Serum cytokine expression in COVID-19 patients.

**Cytokine**	**Normal range**	**Total (*N =* 108)**	**EE group (*N =* 41)**	**PE group (*N =* 67)**	***p*** -value^*^
IL-1β (pg/mL)	0–12.4	1.17 (0.88–1.49)	1.06 (0.72–1.38)	1.24 (1.05–1.59)	0.085
IL-2 (pg/mL)	0–5.71	0.90 (0.58–1.24)	0.76 (0.44–1.21)	0.99 (0.69–1.25)	0.0945
IL-4 (pg/mL)	0–3.0	1.55 (1.19–1.92)	1.39 (1.05–1.98)	1.61 (1.31–1.97)	0.1824
IL-5 (pg/mL)	0–3.1	0.77 (0.65–0.90)	0.70 (0.60–0.80)	0.83 (0.70–0.94)	**0.0023**
IL-6 (pg/mL)	0–5.30	2.09 (1.53–3.09)	1.66 (1.09–3.11)	2.29 (1.78–3.23)	**0.0394**
IL-8 (pg/mL)	0–20.6	2.61 (1.77–3.47)	2.00 (1.41–2.91)	2.75 (2.18–3.53)	**0.0042**
IL-10 (pg/mL)	0–4.91	2.30 (1.73–2.67)	2.12 (1.62–2.75)	2.30 (1.77–2.68)	0.4475
IL-12p70 (pg/mL)	0–3.4	1.16 (0.65–1.59)	1.04 (0.60–1.59)	1.16 (0.64–1.64)	0.4863
IL-17A (pg/mL)	0–20.6	2.06 (1.35–3.44)	1.91 (1.07–3.48)	2.36 (1.63–3.52)	0.5076
TNF-α (pg/mL)	0–4.6	2.05 (1.62–2.55)	2.05 (1.58–2.57)	2.05 (1.62–2.55)	0.7639
IFN-α (pg/mL)	0–8.5	1.11 (0.80–1.45)	0.92 (0.61–1.26)	1.20 (0.86–1.46)	0.061
IFN-γ (pg/mL)	0–7.42	1.27 (1.02–1.55)	1.24 (0.84–1.48)	1.29 (1.13–1.58)	0.1924

Data are presented as median (IQR).

* *p*-value indicates differences between EE and PE patients. *p* < 0.05 is considered statistically significant (in bold).

### Changes in blood routine and blood biochemical test results in patients with COVID-19

The results of blood routine and blood biochemical tests in patients with EE and PE are shown in Table 3. Compared to patients in the EE group, those in the PE group had significantly lower lymphocyte (*p* = 0.0098), eosinophil (*p* = 0.0402), and RBC (*p* = 0.0019) counts; a higher neutrophil count (*p* = 0.0252), and a higher NLR (*p* = 0.0077). Moreover, biochemical blood tests revealed lower concentrations of Hb (*p* = 0.0006) and ALB (*p* = 0.0024) in the PE group than in the EE group.

**Table 3:** Blood routine and blood biochemicals expression in COVID-19 patients.

**Parameters**	**Normal range**	**Total (*N =* 108)**	**EE group (*N =* 41)**	**PE group (*N =* 67)**	***p*** –value^*^
**Blood routine**					
WBC (× 10^9^/L)	3.5–9.5	6.47 (5.42–7.77)	6.80 (5.48–7.77)	6.43 (5.27–7.78)	0.5027
0–3.5		4 (3.70%)	0 (0%)	4 (5.97%)	0.2952
3.5–9.5		98 (90.74%)	38 (92.68%)	60 (89.56%)	0.7390
>9.5		6 (5.56%)	3 (7.32%)	3 (4.48%)	0.6730
Neutrophils (× 10^9^/L)	1.8–6.3	3.86 (3.23–4.75)	3.69 (2.67–4.53)	3.89 (3.38–4.92)	**0.0252**
< 1.8		4 (3.70%)	1 (2.44%)	3 (4.48%)	>0.999
1.8–6.3		98 (90.74%)	38 (92.68%)	60 (89.56%)	0.7390
>6.3		6 (5.56%)	2 (4.88%)	4 (5.97%)	>0.999
Lymphocytes (× 10^9^/L)	1.1–3.2	2.42 (1.47–2.56)	2.42 (1.88–2.93)	1.84 (1.28–2.29)	**0.0098**
< 1.1		15 (13.89%)	2 (4.88%)	13 (19.40%)	**0.0443**
1.1–3.2		85 (78.70%)	33 (80.49%)	52 (77.61%)	0.8115
>3.2		8 (7,41%)	6 (14.63%)	2 (2.99%)	0.0513
Monocytes (× 10^9^/L)	0.1–0.6	0.43 (0.35–0.53)	0.45 (0.39–0.53)	0.43 (0.35–0.53)	0.3934
< 0.1		0 (0%)	0 (0%)	0 (0%)	–
0.1–0.6		92 (85.19%)	34 (82.83%)	58 (86.57%)	0.5917
>0.6		16 (14.81%)	7(17.07%)	9 (13.43%)	0.5917
Eosinophils (× 10^9^/L)	0.02–0.32	0.095 (0.05–0.16)	0.11 (0.07–0.18)	0.08 (0.04–0.15)	**0.0402**
< 0.02		6 (5.55%)	1 (2.44%)	5(7.46%)	0.4123
0.02–0.32		99 (91.67%)	38 (92.68%)	61 (91.04%)	>0.999
>0.32		3 (2.78%)	2 (4.88%)	1 (1.49%)	0.5560
Basophils (× 10^9^/L)	0–0.06	0.02 (0.01–0.03)	0.02 (0.02–0.03)	0.02 (0.01–0.03)	0.7861
≤0.06		107 (99.07%)	41(100%)	66(98.51%)	>0.999
>0.06		1 (0.93%)	0 (0%)	1 (1.49%)	>0.999
NLR		1.95 (1.38–2.83)	1.48 (1.27–2.18)	2.34 (1.54–2.94)	**0.0077**
RBC (× 10^12^/L)	3.8–5.1	4.56 (4.21–4.89)	4.82 (4.43–5.04)	4.43 (4.12–4.76)	**0.0019**
Hb (g/L)	115–150	138 (127–148)	145.5 (132–155.3)	132 (124–146)	**< 0.001**
PLTs (× 10^9^/L)	125–350	235.0 (193.5–280.5)	239 (196–286)	228 (190–278)	0.99
**Blood biochemicals**					
ALB (g/L)	40–55	43.85 (42.08–45.93)	44.7 (42.9–46.6)	43.3 (41.1–45.0)	**0.0024**
ALT (U/L)	7–40	15.50 (11.00–32.25)	16.00 (11.25–35.00)	15.00 (9.75–25.50)	0.1273
AST (U/L)	13–35	23 (19.0–32.0)	23.50 (20.00–33.75)	23.00 (17.75–29.50)	0.1331
ALP (U/L)	50–135	76 (62–89)	82.50 (70.75–90.00)	69.00 (59.75–85.25)	0.2331
GGT (U/L)	7–45	24 (17–34)	21.50(17.00–39.75)	26.50 (15.50–32.00)	0.3906
Total bilirubin (μmol/L)	0–23	9.4 (7.4–12.4)	9.4 (7.7–12.2)	9.25 (6.98–12.88)	0.7275

Data are presented as median (IQR) or n (%).

* *p*-value indicates differences between EE and PE patients. *p* < 0.05 is considered statistically significant (in bold). WBC, white blood cell; NLR, neutrophil-to-lymphocyte ratio; RBC, red blood cell; Hb, hemoglobin; PLTs, platelets; ALB, albumin; ALT, alanine aminotransferase; AST, aspartate aminotransferase; ALP, alkaline phosphatase; GGT, glutamyl transpeptidase.

### Lymphocyte subsets changes in the peripheral blood of patients with non-severe COVID-19

Compared to patients with EE, those with PE had a lower frequency of CD8^+^ T cells (*p* = 0.027) and a higher CD4/CD8 ratio (*p* = 0.0048). In addition, lymphocyte subset counts showed that patients with PE had significantly lower numbers of CD3^+^ T cells (*p* = 0.002), CD4^+^ T cells (*p* = 0.0459), CD8^+^ T cells (*p* = 0.001), and NK cells (*p* = 0.0024) (Table 4).

**Table 4:** Relative frequencies and absolute numbers lymphocyte subpopulations in periphery blood of COVID-19 patients.

**Parameters**	**Normal range**	**Total (*N =* 108)**	**EE group (*N =* 41)**	**PE group (*N =* 67)**	***p*** -value^*^
**Frequencies of lymphocytes (%)**					
B cells	4.7–19.3	12.1 (9.21–16.24)	12.50 (8.64–16.615)	12.06 (9.74–16.20)	0.389
CD3^+^T cells	52.4–81.4	65.57 (60.23–71.11)	64.05 (58.64–70.08)	66.75 (62.36–72.61)	0.444
CD4^+^T cells	23.9–46.3	38.76 (32.11–42.99)	32.82 (30.91–40.74)	39.66 (33.67–44.00)	0.0578
CD8^+^T cells	11.7–40.3	23.31 (19.62–28.03)	25.9 (21.5–29.53)	21.69 (19.05–27.45)	**0.027**
NK cells	8.7–38.3	17.44 (11.44–25.30)	21.17 (14.60–27.39)	15.87 (10.77–23.83)	0.1
**Absolute numbers/**μ**L**					
B cells	102–443	225.9 (172.3–363.4)	266.9 (192.2–394.1)	215.8 (169.5–313.7)	0.282
CD3^+^T cells	948–1,943	1,345 (933–1,758)	1,590(1,148–1,933)	1,222.0 (774.4–1,608.0)	**0.002**
CD4^+^T cells	447–1,030	773.40 (548.5–1,006.8)	894.3 (615.7–1,046.0)	717.4 (507.3–931.9)	**0.0459**
CD8^+^T cells	299–882	487.9 (289.9–641.2)	583.9 (404.9–808.1)	406.9 (254.9–589.7)	**< 0.001**
NK cells	220–735	309.9 (216.3–509.7)	472.6 (288.7–720.6)	251.1 (177.2–409.0)	**0.0024**
CD4/CD8	0.8–3.2	1.58 (1.19–2.13)	1.32 (1.10–1.96)	1.83 (1.24–2.29)	**0.0048**

Data are presented as median (IQR).

* *p*-value indicates differences between EE and PE patients. *p* < 0.05 is considered statistically significant (in bold).

### Correlation between SARS-CoV-2 ORF1ab/N gene Ct values and laboratory findings in patients with non-severe COVID-19

Ct values were negatively correlated with IL-6 (ORF1a/b *p* = 0.0458, N *p* = 0.0426) and IL-8 (ORF1a/b *p* = 0.0208, N *p* = 0.0123) concentrations, and positively correlated with eosinophil count (ORF1a/b *p* = 0.0171, N *p* = 0.01) in patients with COVID-19 (Table 5). The correlation between ORF1ab/N gene Ct values and laboratory findings in EE and PE patients was shown in Supplementary Table S1.

**Table 5:** Correlation between ORF1ab/N gene Ct values and laboratory findings in COVID-19 patients.

	**ORF1ab Ct value**		**N Ct value**	
	***r***	***p-*** **value**	***r***	***p-*** **value**
**Cytokines**				
IL-5	−0.17	0.1072	−0.1435	0.1771
IL-6	−0.2099	**0.0458**	−0.2143	**0.0426**
IL-8	−0.2421	**0.0208**	−0.263	**0.0123**
**Blood routine and blood biochemicals**				
Neutrophils	0.0516	0.6137	0.0181	0.8604
Lymphocytes	0.1739	0.0868	0.1634	0.1097
Eosinophils	0.2403	**0.0171**	0.2603	**0.01**
NLR	0.0058	0.9547	−0.0349	0.7346
RBC	−0.0454	0.6569	−0.0392	0.7032
Hb	0.0885	0.3861	0.0934	0.3628
ALB	0.0801	0.4527	0.0557	0.6039
**Lymphocyte subpopulations**				
% of CD8^+^ T cells	−0.0427	0.6814	−0.0552	0.5955
CD3^+^ T cells numbers	0.0067	0.9487	0.0135	0.897
CD4^+^ T cells numbers	0.0335	0.7471	0.0472	0.65
CD8^+^ T cells numbers	0.0033	0.9744	0.0054	0.9584
NK cells numbers	0.0872	0.401	0.0856	0.4097
CD4/CD8 ratio	0.0581	0.5758	0.0737	0.4776

*p* < 0.05 is considered statistically significant (in bold). ORF, open reading frame; N, nucleocapsid protein.

### Analysis of risk factors for prolonged viral shedding in patients with COVID-19

Finally, we conducted a logistic regression analysis of all 108 patients with COVID-19 to study the risk factors for the viral shedding duration. In univariate logistic regression analysis, 13 variables were included, including sex, age, IL-8, Hb, ALB, NLR, CD4/CD8 ratio, frequency of CD4^+^ T cells, absolute numbers of RBCs, lymphocytes, eosinophils, and NK cells. In multivariate regression analysis, only ALB (*p* = 0.003), CD4/CD8 ratio (*p* = 0.012), NLR (*p* = 0.013), and eosinophil count (*p* = 0.03) were statistically significant (Table 6).

**Table 6:** Univariate and multivariate logistic regression analysis for COVID-19 patients.

**Parameters**	***p*** **-value**	**OR**	**95% CI**
**Univariate analysis**			
Sex	**0.035**	0.420	0.188–0.941
Age	**0.006**	1.039	1.011–1.067
Comorbidity	**0. 005**	0.219	0.076–0. 630
IL-8	**0.030**	1.461	1.038–2.055
Lymphocytes	**0.011**	0.508	0.302–0.855
Eosinophils	**0.048**	0.008	0.000–0.951
NLR	**0.014**	1.686	1.112–2.555
RBC	**0.003**	0.249	0.099–0.627
HB	**0.001**	0.954	0.927–0.981
ALB	**0.005**	0.800	0.686–0.933
% of CD4^+^T cells	**0.022**	1.066	1.009–1.126
NK cells numbers	**0.005**	0.998	0.996–0.999
CD4/CD8 ratio	**0.008**	2.496	1.273–4.892
**Multivariate analysis**			
ALB	**0.003**	0.746	0.614–0.906
CD4/CD8 ratio	**0.012**	3.226	1.296–8.034
NLR	**0.013**	2.407	1.208–4.799
Eosinophils	**0.030**	0.001	0.000–0.502

*p* < 0.05 is considered statistically significant (in bold). OR, odds ratio; CI, confidence intervals.

## Discussion

The SARS-CoV-2 Omicron variant appeared when humanity was on the verge of achieving worldwide immunity through global vaccination against SARS-CoV-2 (14). Compared to the Delta variant, which has 16 mutations, Omicron has approximately 32 mutations in the spike protein (15), making it a public health concern, indicating that this disease pandemic is still far from over (16). In this study, we closely investigated the clinical data and laboratory findings of patients with non-severe COVID-19. We found a significant decline in eosinophil and lymphocyte subsets and an increase in the numbers of IL-6, IL-8, and neutrophils; NLR; and CD4/CD8 ratio in patients with PE. In addition, the Ct values of the ORF1ab/N gene were correlated with IL-6 and IL-8 levels and eosinophil count. However, regression analysis showed that only ALB, NLR, CD4/CD8 ratio, and eosinophil count were risk predictors for the viral shedding duration in patients with non-severe COVID-19.

In total, 108 non-severe SARS-CoV-2 Omicron variant-infected patients were enrolled in this study and allocated to the EE or PE group. Sex, age, multiple symptoms, comorbidities, and coronavirus vaccination status were recorded on admission. Data analysis showed patients with PE were 7 years older and had a higher frequency of comorbidities than those with EE. Moreover, women were more susceptible to prolonged viral shedding than men. There was no difference in COVID-19 vaccination status and most symptoms between the two groups, except for diarrhea. These results indicate that gender, age, and comorbidities, but not symptoms and vaccine inoculation, might be associated with the persistent presence of SARS-CoV-2 in patients with non-severe COVID-19. In addition, sex, age, and ALB were risk factors for prolonged viral elimination in univariate regression analysis. However, only ALB was statistically significant in multivariate regression analysis. Therefore, our results indicated that ALB concentration was a risk factor in viral shedding in patients with non-severe COVID-19.

As demonstrated by antiviral activity, eosinophils participate in adaptive immunity and serve as antigen-presenting cells (17). During influenza A infection, eosinophils promote host cellular immunity by acting as professional antigen-presenting cells and stimulating virus-specific CD8^+^ T cells to reduce influenza virus replication in the lungs (18). In hospitalized COVID-19 patients, eosinopenia was commonly reported (19, 20) and suggested to be an indicator of disease severity (21). In contrast, increased eosinophil counts were correlated with a better prognosis for COVID-19, including a lower incidence of complications and mortality (22). In our study, similar results were found—eosinophil counts were lower in patients with PE than in those with EE and were positively correlated with Ct values of ORF1ab and N genes. Moreover, eosinophils can serve as risk predictors of viral shedding in patients with COVID-19. Our findings suggest that eosinophils are efficient indicators for predicting the viral load and viral shedding duration in patients with non-severe COVID-19.

Leukocytosis, lymphopenia, and a high NLR are the first blood cell changes during SARS-CoV-2 infection (23). Neutrophils are the most abundant immune cells in human blood, accounting for approximately 50–70% of all leukocytes (24), and have been suggested to eradicate the virus through innate immunity (25, 26). During viral infection in the lower respiratory tract, viruses can bind to the surface of epithelial cells, thereby increasing the production of chemoattractant IL-8, which drives the release of neutrophils from the bone marrow (27, 28). IL-8 was one of the earliest and strongest predictors in patients with critical COVID-19 (29), and IL-5 and IL-6 levels were higher in severe COVID-19 cases than in mild ones (30, 31). Our results are consistent with these results, showing that IL-5, IL-6, and IL-8 concentrations and neutrophil counts were more prominent in the PE group than in the EE group, but only IL-6 and IL-8 were negatively correlated with Ct values of ORF1ab and N genes. However, we further enrolled IL-6 and IL-8 in regression analysis and found out that neither IL-6 nor IL-8 was statistically significant in multivariate regression analysis, which suggests that IL-6 and IL-8 weren't effective indicators for viral shedding duration in patients with non-severe COVID-19, and can't reach to the better sensitivity and specificity as compared to the level of nucleic acid.

Similar to patients with severe acute respiratory syndrome or Middle East respiratory syndrome, lymphopenia is commonly observed in patients with COVID-19, and non-survivors develop more severe lymphopenia over time (32–34). In this study, a significant decrease in lymphocyte subsets, including CD3^+^, CD4^+^, and CD8^+^ T cells, and NK cells, was observed in the PE group, indicating that lymphopenia was more notable in persistent virus-positive cases. The adaptive immune response, especially the T-cell response against COVID-19, is critical for mounting resistance against the virus. CD8^+^ T cells played a significant role in controlling viral infection by directly killing virus-infected cells, or producing effector cytokines, including perforin, granzymes, and IFN-γ. In contrast, CD4^+^ T cells assist CD8^+^ T cells and B cells and enhance their ability to clear pathogens (35). Vabret et al. (36) reported that the coordinated action of CD4^+^ and CD8^+^ T cells led to a milder form of the disease, aided by faster viral clearance. In the present study, neutropenia and lymphopenia in patients with PE resulted in a marked increase in NLR. However, the decrease in the number of CD4^+^ T cells was not as substantial as that of CD8^+^ T cells, increasing the CD4/CD8 ratio in patients with PE. This finding is consistent with that reported by Wang et al. (33), who reported that a high CD4/CD8 ratio was an independent predictor for poor clinical efficacy. Moreover, regression analysis showed that both NLR and CD4/CD8 ratios were associated with prolonged elimination of the virus, suggesting NLR and CD4/CD8 ratios as effective indicators of the viral shedding duration in patients with non-severe COVID-19.

Our study has some limitations. First, data collection and laboratory examinations were conducted at a single hospital, which may have resulted in a selection bias. Second, the sample size of non-severe patients enrolled in our study was relatively small, and the sample size might limit the interpretation of our findings. Third, limited laboratory examinations were available for the patients included in this study, and these laboratory findings were not continuously monitored during the course of the disease. Therefore, further studies are needed to investigate the correlation between viral shedding duration and immunity in Omicron variant-infected patients.

## Conclusion

In conclusion, the present study investigated the changes in immune cells, serum cytokines, and blood biochemical parameters in non-severe Omicron variant-infected patients. We found that ALB, NLR, CD4/CD8 ratio, and eosinophil count were effective and efficient predictors of viral shedding duration, which might help discharge management for patients with non-severe COVID-19.

## Data availability statement

The raw data supporting the conclusions of this article will be made available by the authors, without undue reservation.

## Ethics statement

The studies involving human participants were reviewed and approved by the Ethics Committee of Zhongnan Hospital of Wuhan University. Written informed consent from the participants' legal guardian/next of kin was not required to participate in this study in accordance with the national legislation and the institutional requirements.

## Author contributions

WW conceived the original idea of the work. YF and WW collected the data. SH and YF performed, analyzed, interpreted the data, and drafted the manuscript. JY and WW reviewed the manuscript. All authors contributed to the article and approved the submitted version.
