# Supplementary material for: Association between immunity and viral shedding duration in non-severe SARS-CoV-2 Omicron variant-infected patients
Source: Front Public Health. 2022 Dec 22;10:1032957. doi: 10.3389/fpubh.2022.1032957 (PMC9813739; doi:10.3389/fpubh.2022.1032957)
Supplement: Supplementary file 1 [file Table_1.docx]

|  | **EE group** | | | | **PE group** | | | |  |
| --- | --- | --- | --- | --- | --- | --- | --- | --- | --- |
|  | **ORF1a/b Ct value** | | **N Ct value** | | **ORF1a/b Ct value** | | **N Ct value** | |  |
|  | **r** | **p-value** | **r** | **p-value** | **r** | **p-value** | **r** | **p-value** |  |
| **Cytokines** | | | | | | | | | |
| **IL-1β** | -0.035 | 0.8372 | -0.0009 | 0.996 | 0.1859 | 0.1825 | 0.2205 | 0.1126 |  |
| **IL-2** | -0.0746 | 0.6607 | -0.04 | 0.814 | 0.2286 | 0.0996 | 0.2656 | 0.0546 |  |
| **IL-4** | -0.0048 | 0.9773 | 0.00758 | 0.9645 | 0.1568 | 0.2622 | 0.1906 | 0.1717 |  |
| **IL-5** | -0.2089 | 0.2147 | -0.2108 | 0.2105 | 0.205 | 0.1409 | 0.1606 | 0.2506 |  |
| **IL-6** | -0.5189 | **0.001** | -0.5064 | **0.0014** | 0.1752 | 0.2097 | 0.1737 | 0.2136 |  |
| **IL-8** | -0.5457 | **0.0005** | -0.5578 | **0.0003** | 0.08371 | 0.5512 | 0.07395 | 0.5987 |  |
| **IL-10** | -0.3623 | **0.0276** | -0.3478 | **0.0349** | 0.1172 | 0.4031 | 0.1012 | 0.4708 |  |
| **IL-12p70** | -0.169 | 0.3173 | -0.1761 | 0.2973 | 0.144 | 0.3037 | 0.1663 | 0.2339 |  |
| **IL-17A** | -0.1686 | 0.3185 | -0.1409 | 0.4054 | 0.08232 | 0.5579 | 0.0476 | 0.735 |  |
| **TNF-α** | -0.1225 | 0.4701 | -0.088 | 0.6044 | 0.275 | **0.0463** | 0.3148 | **0.0217** |  |
| **IFN-α** | -0.4983 | **0.0017** | -0.4622 | **0.004** | 0.2043 | 0.1422 | 0.1569 | 0.2617 |  |
| **IFN-γ** | -0.3551 | **0.031** | -0.3738 | **0.0227** | 0.07449 | 0.5961 | 0.06129 | 0.6629 |  |
| **Lymphocyte subpopulations** | | | | | | | | | |
| **% of B cells** | 0.2761 | 0.0981 | 0.2716 | 0.1039 | 0.3545 | **0.0068** | 0.3793 | **0.0036** |  |
| **% of T cells** | -0.0679 | 0.6895 | -0.1073 | 0.5273 | -0.0325 | 0.8105 | -0.0374 | 0.7823 |  |
| **% of CD4^+^T Cells** | 0.03014 | 0.8594 | 0.00332 | 0.9844 | 0.06731 | 0.6188 | 0.07689 | 0.5697 |  |
| **% of CD8^+^T Cells** | -0.1378 | 0.416 | -0.146 | 0.3885 | -0.0614 | 0.6501 | -0.0542 | 0.6891 |  |
| **% of NK Cells** | -0.0875 | 0.6064 | -0.0439 | 0.7965 | -0.1424 | 0.2906 | -0.1481 | 0.2715 |  |
| **B cells numbers** | 0.4518 | **0.005** | 0.4374 | **0.0068** | 0.2105 | 0.116 | 0.2192 | 0.1014 |  |
| **T cells numbers** | 0.388 | **0.0177** | 0.3604 | **0.0284** | -0.0671 | 0.6197 | -0.0671 | 0.62 |  |
| **CD4^+^ T cells numbers** | 0.3868 | **0.018** | 0.3572 | **0.03** | -0.0862 | 0.524 | -0.0877 | 0.5165 |  |
| **CD8^+^ T cells numbers** | 0.2923 | 0.0792 | 0.2771 | 0.0968 | -0.0374 | 0.7822 | -0.0317 | 0.815 |  |
| **NK cells numbers** | 0.2262 | 0.1782 | 0.2595 | 0.1209 | -0.1365 | 0.3113 | -0.1437 | 0.2864 |  |
| **CD4/CD8ratio** | 0.1314 | 0.4383 | 0.1242 | 0.4639 | 0.0599 | 0.6581 | 0.06038 | 0.6555 |  |

Table S1

Table S1: Correlation between ORF1ab/N gene Ct values and laboratory findings in EE and PE patients. p＜0.05 is considered statistically significant (in bold).
